# Network of anxiety and depression symptoms in older Chinese adults living alone: a cross-sectional study

**DOI:** 10.3389/fpsyt.2025.1576964

**Published:** 2025-05-28

**Authors:** Wenqin Chen, Xin Zhao, Tao Xu, Linghui Fang, Chen Hong, Yiqian Xie, Weiman Yan, Xiaolu Zhou, Yang He

**Affiliations:** ^1^ School of Education, Shanghai Normal University, Shanghai, China; ^2^ Teacher Education College, Hunan City University, Yiyang, Hunan, China; ^3^ College of Educational Sciences, Xinyang Normal University, Xinyang, Henan, China; ^4^ Department of Psychology, Second Sanatorium of Air Force Healthycare Center for Special Services Hangzhou, Hangzhou, China; ^5^ School of Psychology, Shanghai Normal University, Shanghai, China; ^6^ Manufacturing department, Xi'an Changli Oil & Gas Engineering & Technical Services Co., Xian, Shaanxi, China

**Keywords:** anxiety, depression, living alone, network analysis, older adults

## Abstract

**Background:**

With the rapid aging of China’s population, the proportion of older adults living alone has increased significantly, bringing their mental health concerns into sharp focus. This study aims to explore the network structure of anxiety and depressive symptoms among older Chinese adults who live alone, thereby identifying central and bridging symptoms to provide scientific evidence for potential intervention targets in prevention and treatment.

**Methods:**

A total of 1,952 older Chinese adults, aged 65 and older, living alone, were selected from the Chinese Longitudinal Healthy Longevity Survey (CLHLS) conducted in 2017-2018. We assessed anxiety and depressive symptoms using Generalized Anxiety Disorder Scale (GAD-7) and the Center for Epidemiologic Studies Depression Scale (CES-D). We identified central and bridge symptoms via expected influences (EI) and bridge expected influences (BEI); network stability was evaluated using bootstrap methods.

**Results:**

The network structure uncovered four crucial connections between anxiety and depressive symptoms. GAD4 “Trouble relaxing”, GAD2 “Uncontrollable worry”, and CESD3 “Feeling blue/depressed” exhibited the highest EI values within the network. Meanwhile, GAD1 “Nervousness or anxiety” and CESD10 “Sleep disturbances” showed the highest BEI values within their respective communities.

**Conclusion:**

This exploratory study is the first to examine the reciprocal relationship between depressive and anxiety symptoms in older Chinese adults living alone. Targeting these central and bridging symptoms may effectively prevent comorbidity and facilitate targeted interventions for those at risk or currently experiencing these symptoms.

## 1 Introduction

On a global scale, aging has emerged as an increasingly prominent and undeniable social phenomenon, exerting a profound impact on numerous facets of human society (1). According to the World Health Organization (WHO), individuals aged 60 and above are recognized as older adults, and this segment of the global population is projected to surge by 34%, from 1 billion to 1.4 billion, between 2020 and 2030 (2). China, which boasts the largest older adults population, also experiences one of the fastest aging rates globally (3). Projections indicate that by 2050, the number of Chinese individuals aged 65 and older will reach 400 million, with those aged 80 and above exceeding 150 million (4).

Against this backdrop, the population of older adults living alone has expanded significantly, and the family structure has shifted from the traditional co-living model to a more diversified one (5, 6). In 2015, the population of older adults living alone in China accounted for 13.1% of the total the population of older adults (7). By 2020, the total number of older adults living alone and those in empty-nest families had soared to approximately 118 million (8), suggesting that the number of older adults living alone will continue to climb in the future.

Mental health, a core component of the health system, has garnered increasing attention from society (9). Research reveals that older adults living alone generally exhibit poorer mental health status compared to those living with others (10–12). Comparative studies across East Asia further underscore this disparity. Specifically, recent data indicate that 35.19% of older adults in China suffer from depression (13), and the prevalence soars to 47.8% among those living alone (14). Regionally, a similar pattern emerges: in South Korea, the depression prevalence among older adults living alone is 48.3% (15), while in Japan it is 18% (16). Likewise, anxiety prevalence follows a comparable trend, with 13.41% of Chinese older adults living alone experiencing anxiety (17) and 17.2% of their Korean counterparts affected (18). These cross-national comparisons highlight both the commonalities and culturalspecific differences in mental health challenges faced by aging populations.

Anxiety and depression, the primary forms of mental health disorders, have emerged as key contributors to the escalating global burden of mental health diseases (19, 20). Substantial evidence indicates a high degree of comorbidity between these two conditions. A survey conducted by the World Health Organization revealed that 45.7% of patients with lifelong depression also met the diagnostic criteria for an anxiety disorder (21). This co-occurrence is particularly pronounced in the elderly population. For instance, the prevalence of anxiety and depression comorbidity in the general elderly population in China was 46.74% (22), significantly higher than the 31.6% observed in the younger population (23). Similarly, among older adults in other countries, such as the United States and several European nations, there is ample evidence of high comorbidity between these two psychiatric disorders. Studies have found a comorbidity rate of anxiety and depression of 51.8% in community-dwelling older adults in the United States (24) and 49.3% in the Netherlands (25). The high rates of anxiety and depression comorbidity in older adults across different countries underscore similar patterns in cross-cultural studies.

Among older adults living alone, these two emotional disorders are particularly prominent. Due to the absence of daily family companionship and emotional support, they are more prone to loneliness and helplessness, thereby triggering anxiety and depression (26–30). Notably, compared with older adults living with their spouses, those living alone exhibit higher levels of depressive and anxious symptoms (31, 32). A meta-analysis further confirms that older adults living alone have a significantly elevated risk of developing depression compared to those living with others (33). Moreover, anxiety not only frequently occurs among older adults living alone but may also exacerbate the relationship between their loneliness and cognitive decline, further highlighting its pivotal role in the formation of depressive emotions (34, 35). These issues not only severely undermine the quality of life of the older adults but may also have long-term adverse effects on their physical health and social functions, even increasing the risk of suicide (36–39).

Although the impacts of anxiety and depression on older adults living alone have been widely acknowledged, previous studies have predominantly utilized traditional scale-based assessment methods (40–42). These methods, grounded in the total score model of anxiety or depression scales, primarily focus on evaluating the overall severity of symptoms. However, this approach may overlook the heterogeneity and differential importance among symptoms (43, 44). For instance, anhedonia, a core symptom of depression, holds greater significance than non-core symptoms such as fatigue. In light of this, in-depth analyses of symptom levels are emerging as a new perspective for gaining insight into the complex intertwined relationships between symptoms (45).

In recent years, network analysis, an emerging data-driven approach, has offered a fresh perspective for exploring the structure of psychopathology and the interactions between symptoms (46). Within the framework of network analysis, each symptom is regarded as a node within the network, and the interactions between symptoms constitute the edges of the network, enabling the revelation of concealed interaction relationships and potential connections among symptoms (47). By calculating the centrality index and the bridge centrality index, network analysis can pinpoint the key symptoms in the comorbidity of anxiety and depression. These symptoms are not only crucial for comprehending the comorbidity mechanism but also serve as vital targets for formulating effective prevention and intervention strategies (48, 49).

Despite the increasing application of network analysis in the field of psychology (50), in-depth research on the network of anxiety and depression symptoms in older adults living alone remains scant. This dearth means that when addressing the mental health issues of this large-scale population, we lack sufficient scientific evidence and effective means. Therefore, conducting this study is both urgent and necessary.

Given the aforementioned background, this study aims to bridge this research gap. For the first time, we will employ network analysis to construct a network model of anxiety and depression symptoms among older Chinese adults living alone. Based on network theory principles (46, 47), we hypothesize that specific anxiety symptoms are significantly associated with depressive symptoms; specific symptoms (e.g., excessive worry too much in anxiety, feeling blue/depressed in depression) will act as central nodes with high influence; and bridge symptoms (e.g., sleep disturbances, nervousness or anxiety) will show strong cross-disorder connections between anxiety and depression clusters. Our objective is to explore the fine-grained associations between these two types of symptoms and to identify the key central and bridging symptoms. Our hope is to provide potential targets for the prevention and intervention of anxiety and depression symptoms in this population. By analyzing the relationships between symptoms from a network perspective, we aim to lay a foundation for understanding the mental health of older adults living alone and to guide the development of intervention measures. For instance, based on the identified key symptoms, we can devise targeted psychological counseling programs and design specialized social activities. These measures can alleviate anxiety and depression, improve mental health, and enhance the quality of life and social participation of the older adults. This study holds significant theoretical and practical importance.

## 2 Materials and methods

### 2.1 Participants

This research utilizes the dataset from the seventh wave (2017–2018) of the China Longitudinal Healthy Longevity Survey (CLHLS), a collaborative project led by the Center for Healthy Aging and Development Research and the National School of Development at Peking University. The CLHLS is designed to conduct comprehensive longitudinal studies on the older adults population across 23 provinces, autonomous regions, and municipalities in China. Initiated in 1998, the project has undergone multiple follow-ups, including surveys in 2000, 2002, 2005, 2008-2009, 2011-2012, 2014, and culminating in 2017-2018, with a sample of 15,874 individuals aged 65 and older.

The operational definition of “living alone” was based on Question A5–1 in the CLHLS questionnaire: “With whom do you currently live?” The response options included (1): living with family members (including live - in babysitters) (2); living alone (3); residing in a nursing facility. Participants who selected option 2 (“living alone”) were included in the study, while those choosing options 1 or 3, or providing missing responses, were excluded. This strict criterion ensured that the study population consisted solely of older adults without co-residing family members or institutional caregivers.

**Figure 1:**
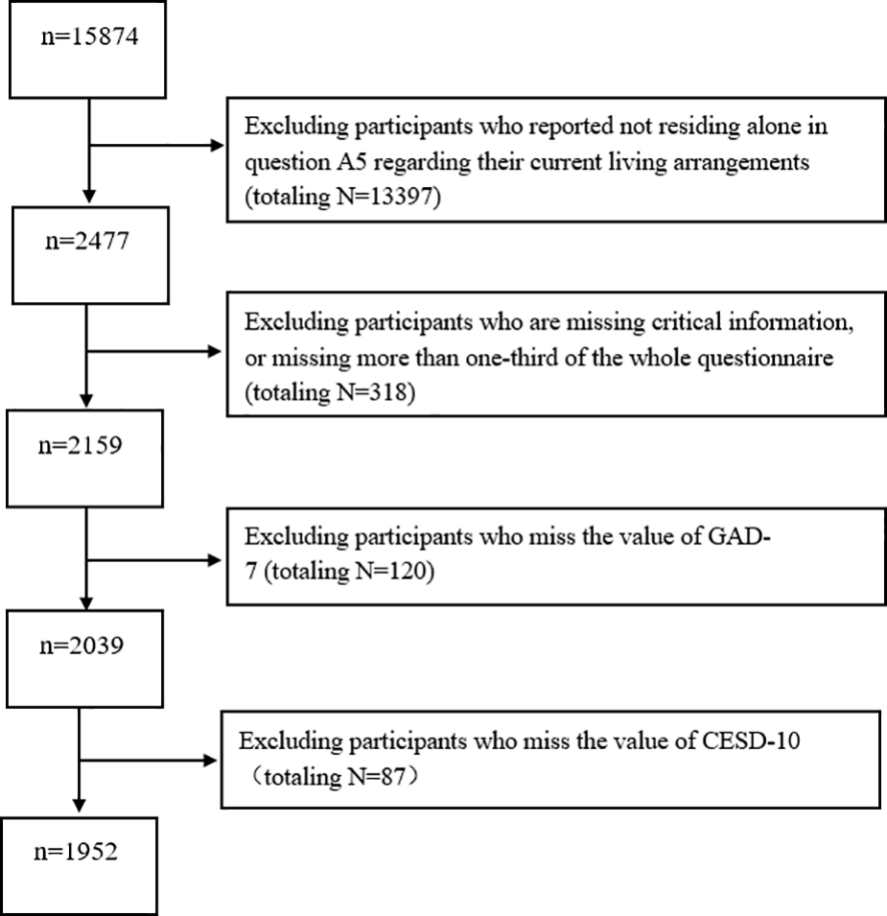
Flow diagram of the sample selection, the Chinese Longitudinal Healthy Longevity Survey (CLHLS 2017-2018).

The primary focus of this research is on older adults within the CLHLS dataset who met the “living alone” criterion (initial sample N = 2,477). To maintain data integrity, a multi -stage screening process was implemented. First, 318 participants were excluded due to missing critical demographic information (e.g., age, gender) or incomplete questionnaires (defined as missing more than one - third of the total items). Subsequent exclusions targeted incomplete mental health assessments: 120 participants lacked valid responses for the Generalized Anxiety Disorder-7 (GAD-7) scale, and 87 participants had missing data for the Center for Epidemiologic Studies Depression Scale-10 (CES-D). After these exclusions, the final analytical sample consisted of 1,952 eligible older adults living alone (see **Figure 1** for the complete selection flowchart). Notably, the CLHLS study design was approved by the Biomedical Ethics Committee of Peking University (N0: IRB00001052-13074), ensuring adherence to ethical guideline.

### 2.2 Measurements

#### 2.2.1 Center for epidemiologic studies depression scale

We used CESD to measure depression symptom, and CESD contains 10 items rated on a Likert scale ranging from 0 “never” to 3 “always” (51). The total CESD scores range from 0 to 30, with higher scores indicating more severe depressive symptoms. According to previous studies, a CESD score ≥ 10 is considered to indicate the presence of depressive symptoms (52). The scale has been widely used in the Chinese older adults (37, 53, 54). The scale has good validity and reliability, in our study, the Cronbach’s α coefficient of the scale was 0.79 and the Kaiser - Meyer - Olkin (KMO) score was 0.82.

#### 2.2.2 Generalized anxiety disorder

We used GAD to assess the severity of anxiety symptoms (55). GAD-7 contains 7 items, and each item has a score from 0 to 3, representing “not at all” to “almost every day”. The total GAD-7 score is between 0 and 21; a higher score indicates more serious anxiety symptoms. Previous studies have confirmed that the GAD-7 has been shown to have good validity and reliability in older adults (37, 53). According to previous studies, a GAD score ≥ 5 is considered to indicate the presence of anxiety symptoms (56). In our study, the Cronbach’s α coefficient of the scale was 0.90, demonstrating excellent internal consistency. The Kaiser - Meyer - Olkin (KMO) score was 0.85, indicating that the data were appropriate for factor analysis.

### 2.3 Data analysis

All descriptive data were analyzed using IBM SPSS Statistics (Version 25.0) software (Armonk, NY: IBM Corp). All network analysis were conducted using R (Version 4.1.2).

#### 2.3.1 Network estimation

We utilized the qgraph and bootnet R packages (57) to construct and conduct a rigorous evaluation of the anxiety-depression network within a cohort of older adults residing in solitary living arrangements. Within the confines of this sophisticated network, blue lines serve as visual cues for positive correlations, whereas red lines symbolize the presence of negative correlations between various manifestations of anxiety and depression. The magnitude of these correlations is vividly represented through the varying thickness of the lines and the saturation levels of the colors (58). The nodes within this intricate network are systematically segregated into two distinct communities: the anxiety community and the depression community, based on their respective origins and characteristics.

To enhance the robustness and interpretability of the network, we employed the Least Absolute Shrinkage and Selection Operator (LASSO) (59) regularization technique in tandem with the Extended Bayesian Information Criterion (EBIC) (60). The optimal LASSO parameter was determined through 10-fold cross-validation, selecting the regularization strength that minimized the mean EBIC across the folds. This iterative process effectively balanced model complexity and predictive accuracy while pruning non-informative edges (58). A sensitivity analysis was conducted to evaluate different EBIC hyperparameter values (γ = 0.1, 0.5, 0.9), which revealed consistent community structures across these settings. Based on prior recommendations for psychological networks (60), a value of γ = 0.5 was retained to strike a balance between edge elimination and theoretical validity. Non-parametric Spearman correlations were used to quantify ordinal symptom associations (61), which is appropriate given the skewed distributions commonly observed in clinical data.

Ultimately, we harnessed the Fruchterman-Reingold algorithm (62) to meticulously arrange and visualize the layout of the network. This sophisticated algorithm ensures that the nodes and edges are displayed in a manner that maximizes clarity, readability, and comprehension, thereby facilitating a nuanced understanding of the complex relationships within the anxiety-depression network among older adults living in solitary circumstances.

#### 2.3.2 Centrality and bridge centrality estimation

We utilized the networktools and bootnet R packages (58) to compute and assess the expected influence (EI) and bridge expected influence (BEI) of nodes within the network. Following established network psychometric protocols (46), the EI for each node was calculated as the sum of the absolute values of edge weights connecting it to all other nodes. Higher EI values indicate greater centrality in maintaining symptom activation and network cohesion (63). The BEI of a node quantifies its role as a bridge between symptom clusters (e.g., anxiety and depression) by representing the summation of edge weights linking it to nodes belonging to different communities. Nodes with high BEI are reported to facilitate cross-community symptom propagation, making them strategic targets for clinical interventions aimed at disrupting comorbid pathways (64).

#### 2.3.3 Network accuracy and stability estimation

To evaluate the accuracy and stability of the network, we utilized the bootnet R package (58). Initially, we bootstrapped the 95% confidence intervals for the edge weights to approximate the precision of the network edges. Subsequently, network stability was assessed using the case-dropping subset bootstrap method, employing a correlation stability coefficient (CS). It is important to note that the CS coefficient should not fall below 0.25; values exceeding 0.50 indicate robust stability and interpretability of the network. Lastly, we conducted significance tests on the edge weights, EIs, and BEIs of different nodes, as well as tests for centrality differences within the network. Notably, 1,000 bootstrap iterations were consistently implemented across all analyses in this study.

## 3 Results

### 3.1 Descriptive statistics

The analysis encompassed a cohort of the 1,952 older people living alone in China. The mean age of the participants was 84.27 ± 9.37 years. Within this population, 38.04% were male, 18.39% reported their health status as self-rated, 34.31% hailed from towns, 47.31% originated from rural areas, and merely 18.38% resided in cities. Notably, 1,396 participants exhibited significant depressive symptoms (71.48%, with CESD scores ≥10), among whom 263 also displayed anxiety symptoms (13.47%, with GAD scores ≥5). **Table 1** presents the mean scores, standard deviations, EIs, and BEIs for items within the anxiety-depression network.

**Table 1:** The means, standard deviations, EIs, and BEIs of variables of anxiety and depression symptoms.

Symptoms	Abb	M	SD	EI	BEI
Anxiety symptoms					
Nervousness or anxiety	GAD1	0.34	0.59	0.87	0.46
Uncontrollable worry	GAD2	0.25	0.53	1.14	0.15
Worry too much	GAD3	0.29	0.59	0.55	0.34
Trouble relaxing	GAD4	0.20	0.49	1.15	0.20
Restlessness	GAD5	0.17	0.47	0.98	0.03
Easily annoyed/irritated	GAD6	0.17	0.45	0.75	0.18
Afraid something terrible might happen	GAD7	0.15	0.44	0.86	0.03
Depression symptoms					
Feeling bothered	CESD1	1.05	0.89	0.76	0.17
Difficulty with concentrating	CESD2	1.47	1.02	0.52	0.03
Feeling blue/depressed	CESD3	1.03	0.87	1.07	0.19
Everything was an effort	CESD4	1.63	1.12	0.72	0.28
Hopelessness	CESD5	1.63	1.18	0.64	0.02
Felt fearful	CESD6	0.89	0.86	0.75	0.16
Lack of happiness	CESD7	1.90	1.30	0.58	0.01
Loneliness	CESD8	1.41	1.12	0.81	0.13
Inability to get going	CESD9	0.79	0.86	1.02	0.08
Sleep disturbances	CESD10	1.47	1.02	0.50	0.33

M, mean; SD, standard deviation; EI, expected influence; BEI, bridge expected influence.

### 3.2 Network estimation

**Figure 2:**
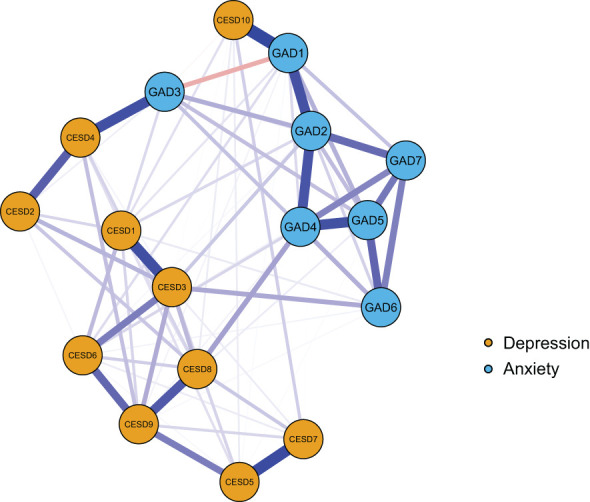
The network model of anxiety and depressive symptoms in older adults living alone. In this network, nodes represent symptoms, and edges represent the correlations between them. The orange nodes indicate depression symptoms, which are assessed using the 10 - item Center for Epidemiologic Studies Depression Scale (CES - D).; the blue nodes indicate anxiety symptoms, measured by the seven - item Generalized Anxiety Disorder scale (GAD-7). The specific meanings of each node are detailed in **Table 1**. The blue lines signify positive partial correlations, whereas the red lines signify negative partial correlations. A thicker line and a more saturated color indicate a larger partial correlation coefficient. The weights of the edges are provided in **Supplementary Table S1**.

The anxiety and depression network model of the older people living alone in China is shown in **Figure 2**. Within this model, there are 23 nonzero edges across the communities (weight range from -0.01 to 0.34). Among these edges, several stand out prominently: the connection between GAD1 “Nervousness or anxiety” and CESD10 “Sleep disturbances” (edge weight = 0.34), the link between GAD3 “Worry too much” and CESD4 “Everything was an effort” (edge weight = 0.28), the correlation between GAD4 “Trouble relaxing” and CESD8 “Loneliness” (edge weight = 0.13), and the association between GAD6 “Easily annoyed/irritated” and CESD3 “Feeling blue/depressed” (edge weight= 0.12). For a detailed overview of the edge weights within this network, please refer to **Supplementary Table S1** in the supplementary materials.

### 3.3 Central symptoms and bridge symptoms

**Figure 3:**
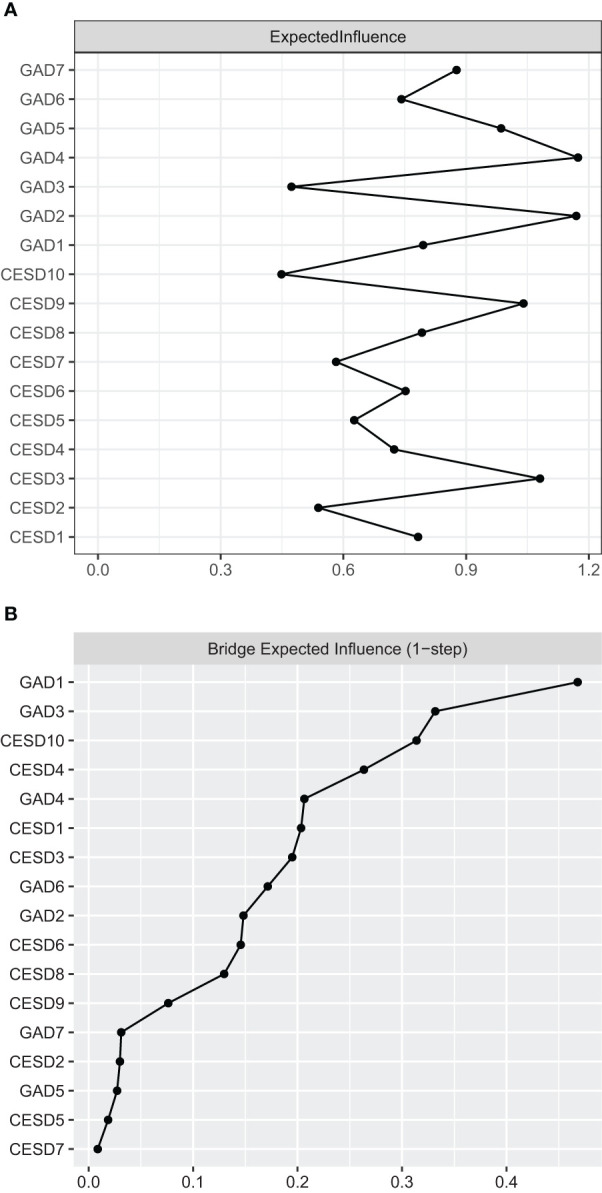
The EIs and BEIs of the nodes in the network. **(A)** The EI indices of the nodes in the network (raw values) and the specific meanings of each node are shown in **Table 1**; **(B)** The BEI indices of the nodes in the network (raw values) and the specific meanings of each node are shown in **Table 1**.

**Figure 3A** shows the EI indices of each node to assess their relative importance in the network. Among them, GAD4 “Trouble relaxing” and GAD2 “Uncontrollable worry” had the highest expected influence (EI = 1.15 and 1.14, respectively), and CESD3 “Feeling blue/depressed” was also statistically stronger than most other nodes in the depression and anxiety network (EI =1.07). This indicates that these three were the most influential symptoms.

**Figure 3B** shows the raw BEI values of each node. In the anxiety community, the nodes GAD1 “Nervousness or anxiety” (BEI = 0.46). In the depression community, the nodes CESD10 “Sleep disturbances” had the highest BEI values (BEI = 0.33). Therefore, these nodes represent critical bridge symptoms in the anxiety-depression network.

### 3.4 Network accuracy and stability

As shown in **Supplementary Figure S1**, the 95% CI derived from the bootstrapping method is narrow, highlighting the high precision and robustness of our edge weight estimates. **Supplementary Figure S2** showcases the results of the bootstrapped difference test for the edge weights, clearly indicating that the weights of the four strongest edges stood out significantly above those of the other nodes. The CS-coefficients of EI and BEI were both 0.75, indicating that our estimates for both EI and BEI were remarkably stable (see **Supplementary Figures S3** and **Supplementary Figures S4**).

The bootstrapped difference test results for node EIs revealed that the EI values of the three central nodes towered over those of the other nodes (see **Supplementary Figure S5**). Furthermore, **Supplementary Figure S6** showcases the findings of the bootstrapped difference test for node BEIs, emphasizing that the BEI values of the bridge nodes are significantly elevated compared to those of the other nodes.

## 4 Discussion

In this study, we constructed a network model to explore the fine-grained relationship between anxiety and depressive symptoms among older Chinese adults living alone. By estimating the EI and BEI values, we identified potential intervention targets and provided recommendations for enhancing the mental health of this population. Notably, this study is the first specifically conducted on older Chinese adults living alone, and as such, it is exploratory in nature, offering only preliminary insights into this field.

### 4.1 The fine-grained relationships between anxiety and depression

In the network model, cross-community edges uncovered complex and subtle associations between anxiety and depressive symptoms. These associations are pivotal for understanding the mechanisms underlying comorbidity and may reflect potential interaction patterns between the two symptoms among older adults living alone (65–67). By focusing on the core cross-community edges of the network model, we discovered that the most significant correlation was between GAD1 “nervousness or anxiety” and CESD10 “sleep disturbance,” which served as a crucial bridging symptom connecting different symptom clusters of anxiety and depression. This finding aligns with previous studies (68, 69). Nervousness or anxiety, as a hallmark anxiety symptom, is particularly prevalent among older adults living alone (70). Due to their prolonged lack of family companionship and social support, they are highly susceptible to such negative emotions when confronted with life’s uncertainties (71). From a physiological psychology perspective, anxiety can disrupt the endocrine system, increasing the secretion of stress hormones like adrenaline, activating the nervous system, and ultimately interfering with the normal regulation of the sleep-wake cycle (72–74). For instance, a recent study on the mental health of older adults within the community revealed that those with elevated anxiety levels were more prone to sleep disorders such as difficulty falling asleep, light sleep, and vivid dreaming (75). This observation concurs with the findings of the present study in a cohort of older adults who live alone, further substantiating the prevalent link between anxiety and sleep disorders (76–78).

In addition, there was a significant positive correlation between GAD3 “Worry too much” and CESD4 “Everything was an effort”, which may indicate a strategic psychological response employed by older adults living alone in coping with life stresses (79). These adults not only confront the challenge of independently managing daily tasks such as shopping, medical appointments, and household chores, but they also grapple with the uncertainty of their health status and profound concerns about their future lives. These stressors can readily plunge them into a state of excessive worry (80). Excessive worrying not only directs cognitive resources excessively toward negative events but also depletes substantial mental energy, consequently weakening executive functioning and rendering even routine daily activities arduous (81). Building upon the theoretical frameworks of cognitive psychology and psychopathology, this finding aligns with the prevalent pattern of anxiety adversely impacting an individual’s daily functioning (82), despite the existing research gap specifically targeting older adults living alone. For instance, prior research on individuals with anxiety disorders has demonstrated that excessive worrying appreciably impairs attention, memory, and decision-making abilities, thereby disrupting daily life and work performance (83–85). Consequently, older adults living alone may endeavor to seek a sense of security and control by redoubling their efforts. However, the efficacy of this coping strategy remains to be further validated, and it may even exacerbate depressive symptoms. This underscores the intricacy of mental health issues among older adults living alone and necessitates more in-depth exploration by researchers.

Meanwhile, there is a strong correlation between GAD4 “Trouble relaxing” and CESD8 “Loneliness”. Due to the scarcity of social interaction, loneliness has become a pervasive aspect of everyday life for older adults living alone (32). This prolonged state of loneliness may perpetuate their psychological state of stress, making it difficult to attain a state of relaxation (86). Prior research has revealed that older adults with chronic physical ailments often experience anxiety, restlessness, and difficulty relaxing when discussing their feelings of loneliness (87). Neurobiological studies have also emphasized the ability of loneliness to disrupt neurotransmitter secretion within the brain, leading to imbalances that interfere with the normal regulation of the nervous system and, subsequently, exacerbating an individual’s struggle to achieve relaxation (88, 89). Furthermore, older adults who experience loneliness exhibit a higher prevalence of anxiety compared to those who do not feel lonely (90). Therefore, the correlation between these two symptoms is readily apparent.

We found that GAD6 “Easily annoyed/irritable” was positively correlated with CESD3 “Feeling blue/depressed” consistent with previous studies (91). Irritability, a typical emotional characteristic of anxiety and depression comorbidity, is particularly pronounced in this population due to their lack of emotional support and social activities, making them more susceptible to irritability when facing life challenges (92, 93). According to role theory, the social role transitions undergone by older adults living alone, coupled with the accompanying uncertainty, increase their risk of experiencing negative emotions (94, 95). Over time, the accumulation of these negative emotions may gradually evolve into a melancholic or depressive state of mind (96). Research has confirmed the reciprocal relationship between anxiety and depressive symptoms (97). For instance, a longitudinal study revealed that older adults with higher initial levels of irritability were more likely to exhibit depressive symptoms at follow-up, with depression severity closely linked to the duration and intensity of their preexisting irritability (98). This underscores that, among older adults living alone, irritability serves not only as an early warning sign for depression but also as a pivotal factor in the interplay between anxiety and depression, further highlighting the unique impact of solitary living on their mental health (93).

### 4.2 The central and bridge symptoms between anxiety and depression

Our network stability analysis revealed high robustness, with a CS of 0.75 for both the EI and BEI indices, which exceeds the recommended threshold of 0.5 (64). This finding indicates that the identified central and bridge symptoms are statistically reliable and not artifacts of sampling variability. Based on EI values demonstrated that “difficulty relaxing” (GAD4) was the most central node impacting the mental health of older adults living alone, closely followed by “uncontrollable worry” (GAD2) and “feeling blue/depressed” (CESD3), all exhibiting high EIs. This suggests that they occupy a pivotal position within the network of anxiety and depressive symptoms in this population. These findings align with previous network analyses of anxiety and depressive symptoms conducted across diverse populations, such as disabled elderly individuals, elderly individuals with diabetes, those with functional impairments, epilepsy, disabled adults, adolescents, and nurses. Consistently, these studies have demonstrated that “difficulty relaxing,” “ uncontrollable worry,” and “feeling blue/depressed” exhibit high centrality indices within the studied populations (56, 70, 101, 102)

Specifically, “Difficulty relaxing” (GAD4) reflects a persistent state of psychological tension among older adults living alone, stemming from social isolation and emotional support deficiencies. This state may serve as a key catalyst in the vicious cycle between anxiety and depressive symptoms, and its alleviation could be of significant importance for enhancing overall mental health (99). Conversely, “uncontrollable worry” (GAD2) exacerbates the psychological burden on these individuals, with death-related anxiety particularly intensifying their psychological distress (100). “Feeling blue/frustrated” (CESD3), as a core depression manifestation, directly diminishes the quality of life and mental well-being of older adults living alone (101). Although similar symptoms have been pinpointed as key central symptoms in studies involving diverse populations (68, 69, 91, 102, 103), the present study underscores that, in the context of older adults living alone, the manifestation and mechanism of action of these symptoms vary according to their unique living circumstances and psychological states, often tied to livelihood insecurity, unmet emotional needs, prolonged loneliness, and inadequate social support (93).

BEI value analyses further illuminated bridging symptoms within the anxiety and depressive symptom network. Notably, “nervousness or anxiety” (GAD1) in the anxiety group exhibited the highest BEI values, aligning with prior findings in depression and anxiety networks among older diabetic patients (69, 101), emphasizing its centrality in anxiety states and strong associations. For older adults living alone, interventions specifically targeting “nervousness or anxiety” (GAD1) may prove more effective in mitigating their depression risk. In the depressed group, “sleep disturbance” (CESD10) possessed the highest BEI value, consistent with previous research (69), indicating its prevalence in depressive symptoms and strong correlation with depression (104). For this population, sleep disturbance is not merely a common depression symptom but may also function as a pivotal factor exacerbating depression levels and intertwine with anxiety symptoms, collectively sustaining the intricate association between anxiety and depression (105).

### 4.3 Clinical implications and intervention strategies

The central and bridging symptoms identified in this study hold significant clinical implications. According to psychopathological network theory, interventions targeting these key symptoms may effectively prevent and treat depression and anxiety among older adults living alone (63, 64). Cognitive behavioral therapy (CBT) has been widely recognized as an effective treatment for anxiety and depression interventions (106).

For specific symptoms, targeted CBT components can be applied. In the case of the central symptom “Trouble relaxing” (GAD4), CBT programs should incorporate combined relaxation training. Evidence shows that such techniques can significantly reduce anxiety levels in older adults, and their effects can last for 14–24 weeks post-intervention (107). Regarding “Worry too much” (GAD3), cognitive restructuring for catastrophic thinking patterns should be prioritized. For example, challenging beliefs like “if I don’t control my worry, terrible things will happen” through Socratic Questioning can facilitate cognitive change and alleviate depressive symptoms (108). For “Feeling blue/depressed” (CESD3), behavioral activation (BA) is necessary. Increasing positively reinforcing behaviors, such as engaging in graded structured daily tasks (starting with a 10-minute walk and progressing to community gardening), can lead to more positive outcomes for individuals and thus improve their mood. A meta-analysis has demonstrated an effect size of 0.72 for BA on late-life depression (109).

For bridging symptoms, corresponding effective interventions also exist. Regarding “sleep disturbance” (CESD10), a meta-analysis showed that Cognitive and Behavioral Therapy for Insomnia (CBT-I) has a 32% response rate in cases of co-morbid insomnia - depression, which is significant for treating both conditions (110). Notably, CBT for sleep disorders has been demonstrated to alleviate the burden of psychiatric disorders and thus enhance mental health (111, 112). As for “nervousness or anxiety” (GAD1), Mindfulness-Based Stress Reduction (MBSR) can break the anxiety-depression cycle through non-judgmental experience and acceptance of the current state of mind. A meta-analysis revealed an effect size of 0.63 for anxiety reduction and 0.59 for depression after using this therapy (113). Additionally, relaxation training and social support interventions can serve as adjunctive treatments to assist older adults living alone in reducing psychological stress and improving their mental wellbeing (107, 114). In particular, addressing “Loneliness” (CESD8) through social support interventions (e.g., group activities, community support) may be particularly beneficial for this population (115).

### 4.4 Limitations and future directions

This study has several limitations. First, the study sample was confined to older Chinese adults living alone, potentially limiting the generalizability of the findings to other populations. Further validation is required to assess the applicability of these results to older adults in different regions or living statuses. Second, the cross-sectional design of the study precluded the establishment of a causal relationship between anxiety and depressive symptoms. Therefore, future longitudinal studies are needed to delve deeper into the dynamic interplay between these two conditions.

Third, reliance on self-report scales introduces potential biases, including social desirability bias, recall inaccuracies, and subjective interpretation variability. The absence of clinical diagnostic validation for anxiety and depression further limits the precision of symptom assessment. Four, in the network construction process, certain covariates or confounders that may impact anxiety and depression in older adults living alone, such as physical health status, economic standing, and social support networks, were omitted. These factors should be comprehensively considered in future research endeavors. Thus, future studies should integrate multi-dimensional data (e.g., physiological indicators, socioeconomic indices) to further validate the specificity of the symptom network.

Lastly, the network structure constructed in this study solely captures group-level average effects and overlooks individual differences and specific psychopathological processes. Group-level analysis inherently obscures individual heterogeneity, as the estimated associations may not reflect person-specific symptom interactions, particularly in this vulnerable subgroup. To address this, future studies might employ more granular research methodologies, such as individual network analysis, to gain a deeper understanding of the relationship between anxiety and depression at the individual level among older adults living alone.

## 5 Conclusion

This study is the first to apply symptom-level network analysis to investigate anxiety and depression symptoms in older Chinese adults living alone. The results revealed that “difficulty relaxing,” “uncontrollable worry,” and “feeling blue/depressed” were the core symptoms, while “nervousness or anxiety” and “sleep disturbance” served as the key bridging symptoms in the anxiety-depression network. These findings offer a crucial foundation for understanding the comorbid mechanisms of anxiety and depression in older adults living alone, as well as suggesting a new direction for developing targeted intervention strategies. Future research should further validate the intervention goals proposed in this study. By leveraging clinical practice and employing more precise research methods, future endeavors should delve deeper into the relationship between anxiety and depression in this population, ultimately providing stronger support for improving the mental health status of olde adults living alone.

## Data Availability

The datasets presented in this study can be found in online repositories. The names of the repository/repositories and accession number(s) can be found below: Publicly available datasets were analyzed in this study. The datasets utilized can be accessed here: https://opendata.pku.edu.cn/dataset.xhtml?persistentId=doi:10.18170/DVN/WBO7LK.
