# Supplementary material for: Network of anxiety and depression symptoms in older Chinese adults living alone: a cross-sectional study
Source: Front Psychiatry. 2025 May 28;16:1576964. doi: 10.3389/fpsyt.2025.1576964 (PMC12153446; doi:10.3389/fpsyt.2025.1576964)
Supplement: Supplementary file 1 [file DataSheet1.docx]

**Supplementary Material**

Supplementary Table S1. The edge weight values in the anxiety-depression network of the older adults living alone in China.

|  | GAD1 | GAD2 | GAD3 | GAD4 | GAD5 | GAD6 | GAD7 | CESD1 | CESD2 | CESD3 | CESD4 | CESD5 | CESD6 | CESD7 | CESD8 | CESD9 | CESD10 |
| --- | --- | --- | --- | --- | --- | --- | --- | --- | --- | --- | --- | --- | --- | --- | --- | --- | --- |
| GAD1 |  | 0.27 | -0.11 | 0.05 | 0.10 | 0.01 | 0.08 | 0.04 | 0.00 | 0.01 | 0.00 | 0.01 | 0.05 | 0.00 | 0.00 | 0.02 | 0.34 |
| GAD2 | 0.27 |  | 0.11 | 0.26 | 0.09 | 0.06 | 0.20 | 0.05 | 0.00 | 0.07 | 0.00 | 0.00 | 0.01 | 0.00 | 0.00 | 0.00 | 0.00 |
| GAD3 | -0.11 | 0.11 |  | 0.08 | 0.09 | 0.00 | 0.04 | 0.05 | 0.02 | 0.00 | 0.28 | 0.00 | 0.00 | 0.00 | 0.00 | 0.00 | -0.01 |
| GAD4 | 0.05 | 0.26 | 0.08 |  | 0.28 | 0.11 | 0.16 | 0.00 | 0.00 | 0.00 | 0.00 | 0.00 | 0.06 | 0.00 | 0.13 | 0.02 | 0.00 |
| GAD5 | 0.10 | 0.09 | 0.09 | 0.28 |  | 0.21 | 0.18 | 0.00 | 0.00 | 0.00 | 0.00 | 0.00 | 0.00 | 0.00 | 0.00 | 0.03 | 0.00 |
| GAD6 | 0.01 | 0.06 | 0.00 | 0.11 | 0.21 |  | 0.17 | 0.03 | 0.00 | 0.12 | 0.00 | 0.00 | 0.02 | 0.00 | 0.00 | 0.01 | 0.00 |
| GAD7 | 0.08 | 0.20 | 0.04 | 0.16 | 0.18 | 0.17 |  | 0.00 | 0.00 | 0.00 | 0.00 | 0.01 | 0.02 | 0.00 | 0.00 | 0.00 | 0.00 |
| CESD1 | 0.04 | 0.05 | 0.05 | 0.00 | 0.00 | 0.03 | 0.00 |  | 0.06 | 0.29 | 0.00 | 0.00 | 0.09 | 0.00 | 0.04 | 0.06 | 0.04 |
| CESD2 | 0.00 | 0.00 | 0.02 | 0.00 | 0.00 | 0.00 | 0.00 | 0.06 |  | 0.10 | 0.23 | 0.00 | 0.02 | 0.00 | 0.08 | 0.00 | 0.00 |
| CESD3 | 0.01 | 0.07 | 0.00 | 0.00 | 0.00 | 0.12 | 0.00 | 0.29 | 0.10 |  | 0.02 | 0.06 | 0.17 | 0.03 | 0.09 | 0.11 | 0.00 |
| CESD4 | 0.00 | 0.00 | 0.28 | 0.00 | 0.00 | 0.00 | 0.00 | 0.00 | 0.23 | 0.02 |  | 0.04 | 0.00 | 0.00 | 0.06 | 0.09 | 0.00 |
| CESD5 | 0.01 | 0.00 | 0.00 | 0.00 | 0.00 | 0.00 | 0.01 | 0.00 | 0.00 | 0.06 | 0.04 |  | 0.01 | 0.31 | 0.00 | 0.17 | 0.03 |
| CESD6 | 0.05 | 0.01 | 0.00 | 0.06 | 0.00 | 0.02 | 0.02 | 0.09 | 0.02 | 0.17 | 0.00 | 0.01 |  | 0.03 | 0.07 | 0.21 | 0.00 |
| CESD7 | 0.00 | 0.00 | 0.00 | 0.00 | 0.00 | 0.00 | 0.00 | 0.00 | 0.00 | 0.03 | 0.00 | 0.31 | 0.03 |  | 0.07 | 0.06 | 0.07 |
| CESD8 | 0.00 | 0.00 | 0.00 | 0.13 | 0.00 | 0.00 | 0.00 | 0.04 | 0.08 | 0.09 | 0.06 | 0.00 | 0.07 | 0.07 |  | 0.24 | 0.03 |
| CESD9 | 0.02 | 0.00 | 0.00 | 0.02 | 0.03 | 0.01 | 0.00 | 0.06 | 0.00 | 0.11 | 0.09 | 0.17 | 0.21 | 0.06 | 0.24 |  | 0.01 |
| CESD10 | 0.34 | 0.00 | -0.01 | 0.00 | 0.00 | 0.00 | 0.00 | 0.04 | 0.00 | 0.00 | 0.00 | 0.03 | 0.00 | 0.07 | 0.03 | 0.01 |  |

**
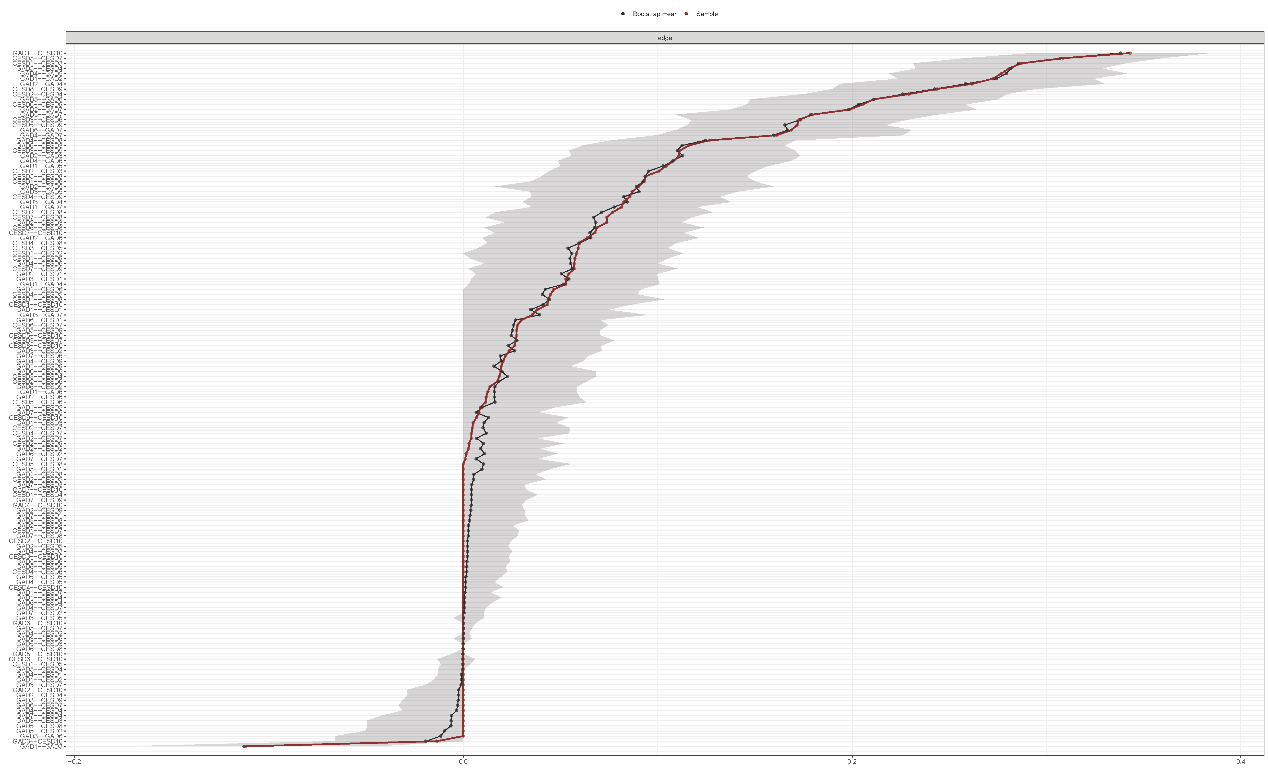
**Supplementary Figure S1 Accuracy of edge weights in the network.

Note: The red line depicts the sample edge weights and the gray bar depicts the bootstrapped confidence interval.


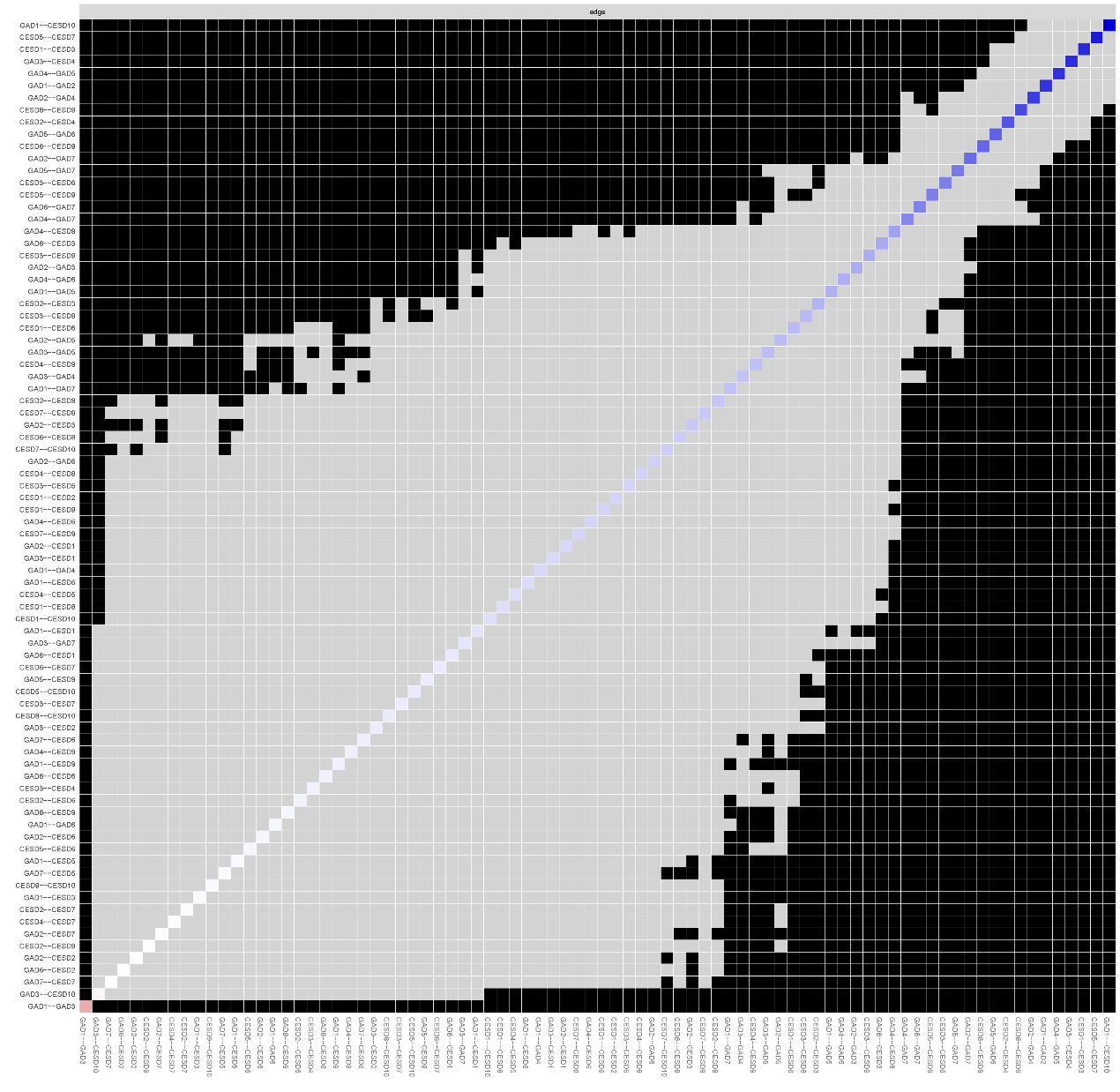


Supplementary Figure S2 Bootstrapped difference test for edge weights in the network.

Note: Gray boxes indicate edge weights that do not differ significantly from one another, while black boxes indicate edge weights that do differ significantly. Blue and red boxes on the diagonal correspond to edge weights with positive and negative correlations, respectively.


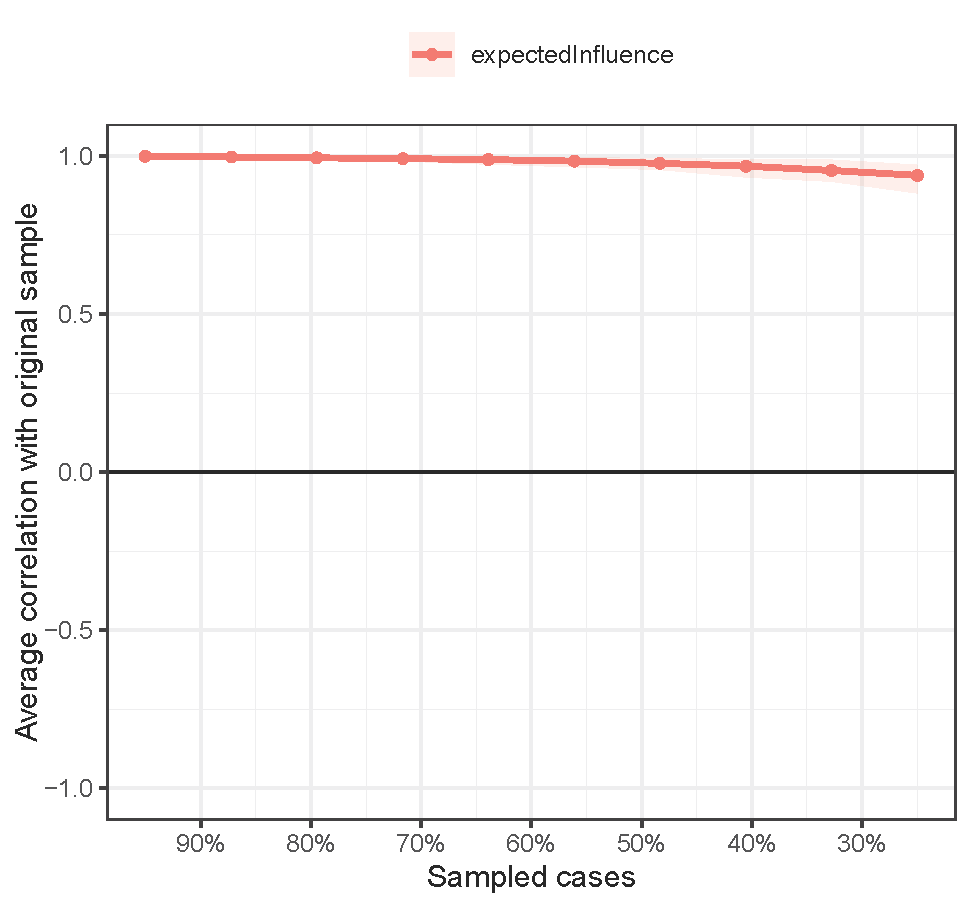


Supplementary Figure S3 Stability of node expected influences in the network.

Note: The red bar represents the average correlation between node expected influences in the full sample and subsample with the red area depicting the 2.5th quantile to the 97.5th quantile.


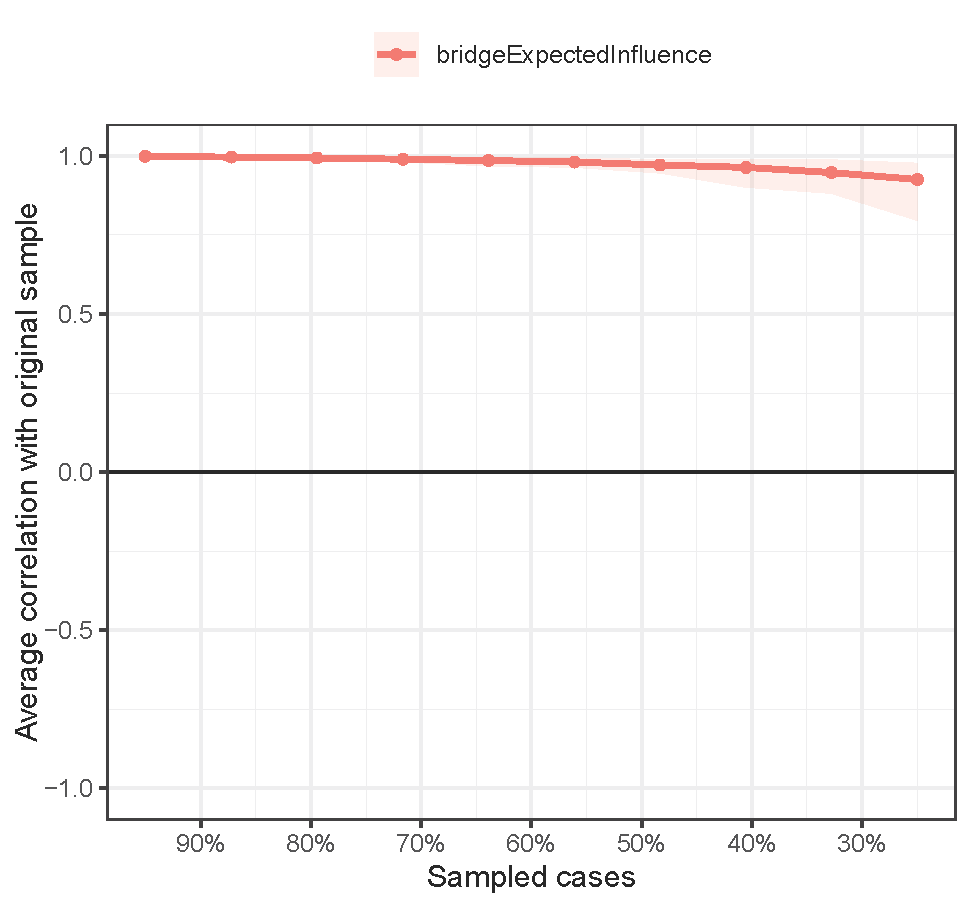


Supplementary Figure S4 Stability of node bridge expected influences in the network.

Note: The red bar represents the average correlation between node bridge expected influences in the full sample and subsample with the red area depicting the 2.5th quantile to the 97.5th quantile.


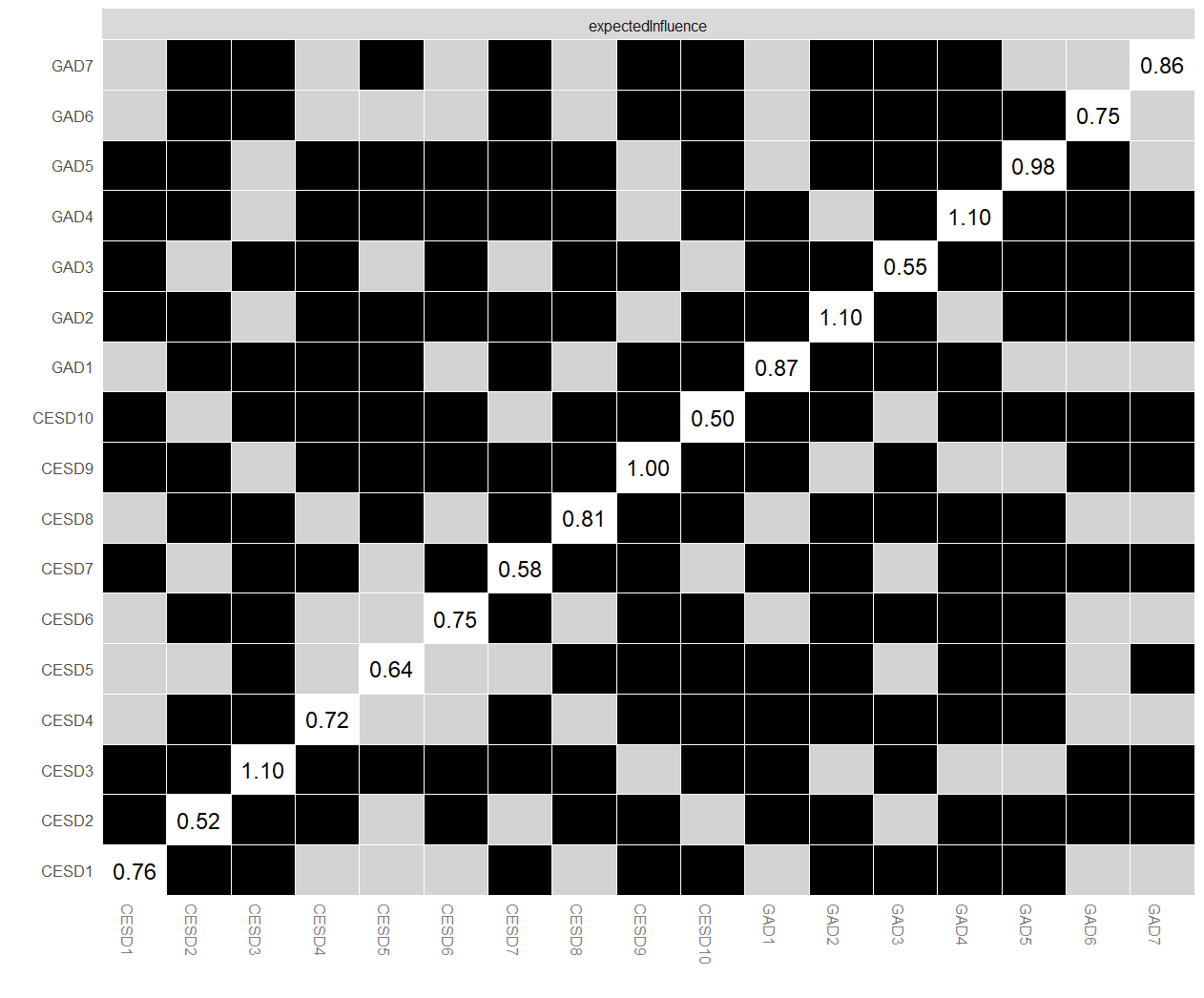


Supplementary Figure S5 Bootstrapped difference test for node expected influences in the network.

Note: Gray boxes indicate node bridge expected influences that do not differ significantly from one another, while black boxes indicate node expected influences that do differ significantly. The numbers in the white boxes (i.e., diagonal line) represent the values of node bridge expected influences.


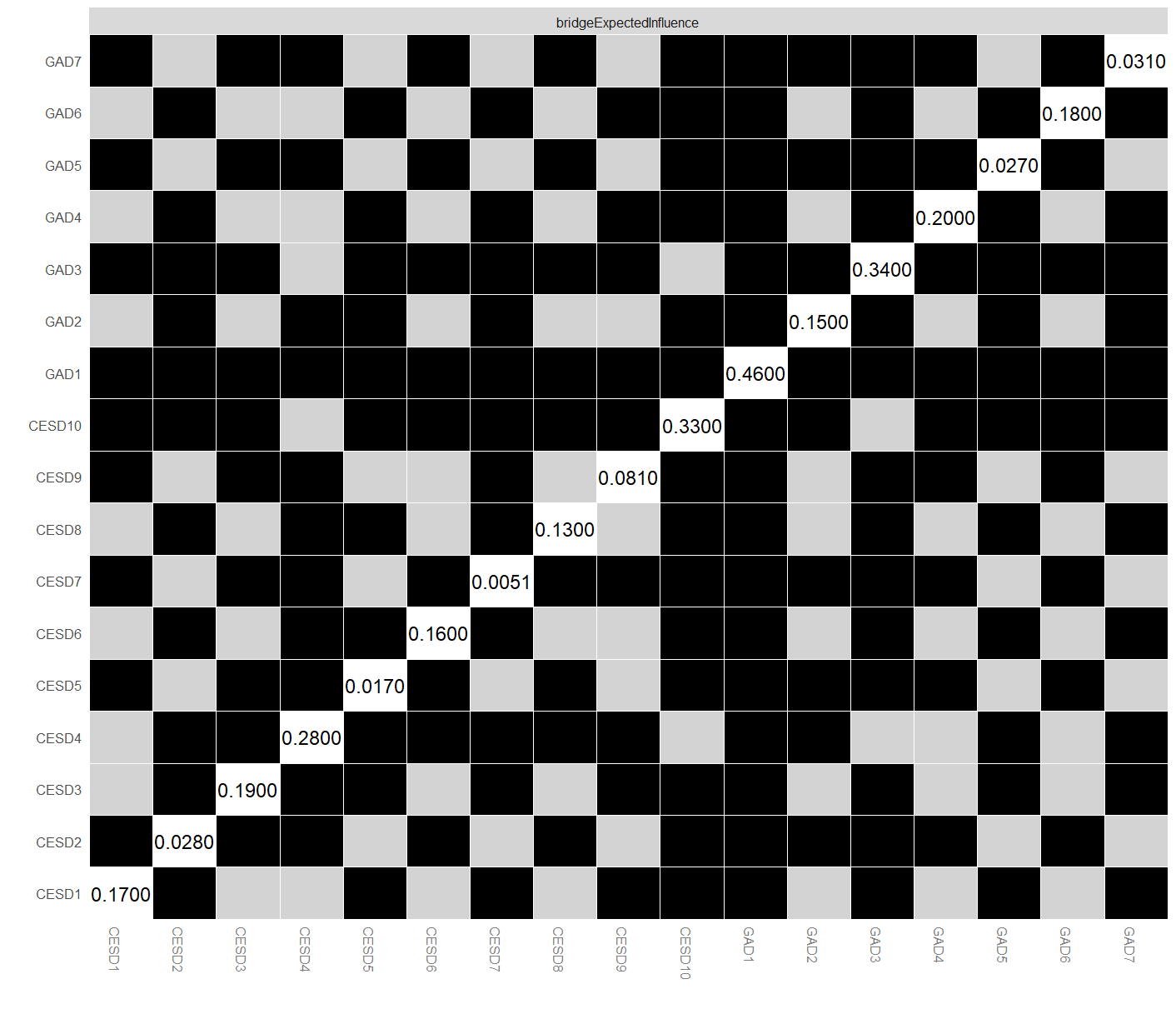


Supplementary Figure S6 Bootstrapped difference test for node bridge expected influences in the network.

Note: Gray boxes indicate node bridge expected influences that do not differ significantly from one another, while black boxes indicate node bridge expected influences that do differ significantly. The numbers in the white boxes (i.e., diagonal line) represent the values of node bridge expected influences.
